# The genome sequence of the acorn piercer, *Pammene fasciana* (Linnaeus, 1761)

**DOI:** 10.12688/wellcomeopenres.18114.2

**Published:** 2024-10-17

**Authors:** Douglas Boyes, University of Oxford and Wytham Woods Genome Acquisition Lab, Darwin Tree of Life Barcoding collective, Wellcome Sanger Institute Tree of Life programme, Wellcome Sanger Institute Scientific Operations: DNA Pipelines collective, Tree of Life Core Informatics collective, Thomas Lewin, Darwin Tree of Life Consortium

**Affiliations:** 1 UK Centre for Ecology and Hydrology, Wallingford, Oxfordshire, UK; 2 University of Oxford, Oxford, Oxfordshire, UK

**Keywords:** Pammene fasciana, acorn piercer, genome sequence, chromosomal, Lepidoptera

## Abstract

We present a genome assembly from an individual male *Pammene fasciana* (acorn piercer; Arthropoda; Insecta; Lepidoptera; Tortricidae). The genome sequence is 564 megabases in span. Most of the assembly (99.94%) is scaffolded into 28 chromosomal pseudomolecules with the Z sex chromosome assembled. The complete mitochondrial genome was also assembled and is 16.4 kilobases in length. Gene annotation of this assembly on Ensembl identified 21,224 protein-coding genes.

## Species taxonomy

Eukaryota; Metazoa; Ecdysozoa; Arthropoda; Hexapoda; Insecta; Pterygota; Neoptera; Endopterygota; Lepidoptera; Glossata; Ditrysia; Tortricoidea; Tortricidae; Olethreutinae; Grapholitini; *Pammene*; *Pammene fasciana* (Linnaeus, 1761) (NCBI:txid1101027).

## Background

*Pammene fasciana*, commonly known as the acorn piercer or the chestnut leafroller, is a moth of the family Tortricidae. *P. fasciana* is univoltine, and is found on the wing in the UK from June to August ( Bland *et al.,* 2015). Adults have a wingspan of 13 to 17 mm, with dark brown forewings, chocolate brown hindwings and a white or ochreous dorsal patch that forms a thick, curved fascia. It is distributed across Europe and into Russia, and mainly inhabits oak woodlands ( GBIF, 2022; Bland *et al.,* 2015). In the British Isles, it is most common in southern England, Wales and the south of the Republic of Ireland, but is also found more sporadically in northern England, Scotland and Northern Ireland ( Bland *et al.,* 2015).

Several tortricid species are among the most economically important pests in Europe and beyond ( Bradley *et al.,* 1979; Kadoić Balaško *et al.,* 2020; Suckling & Brockerhoff, 2010; van der Geest & Evenhuis, 1991), and *P. fasciana* is a notable pest of oak ( *Quercus* spp.) and sweet chestnut ( *Castanea sativa*), especially in mainland Europe ( Avtzis, 2012; Clausi *et al.,* 2016; Speranza, 1998). Eggs are laid on these species’ leaves in the summer; carpophagous (fruit-feeding) larvae hatch approximately two weeks later, and burrow into acorns or chestnuts and consume the kernel, causing premature dropping of fruits, reduced produce quality and subsequently reduced yields and economic losses ( Bland *et al.,* 2015; Pedrazzoli *et al.,* 2012; Speranza, 1998).

The genome of the acorn piercer, *Pammene fasciana*, was sequenced as part of the Darwin Tree of Life Project, a collaborative effort to sequence all named eukaryotic species in the Atlantic Archipelago of Britain and Ireland.

## Genome sequence report

**Figure 1.**
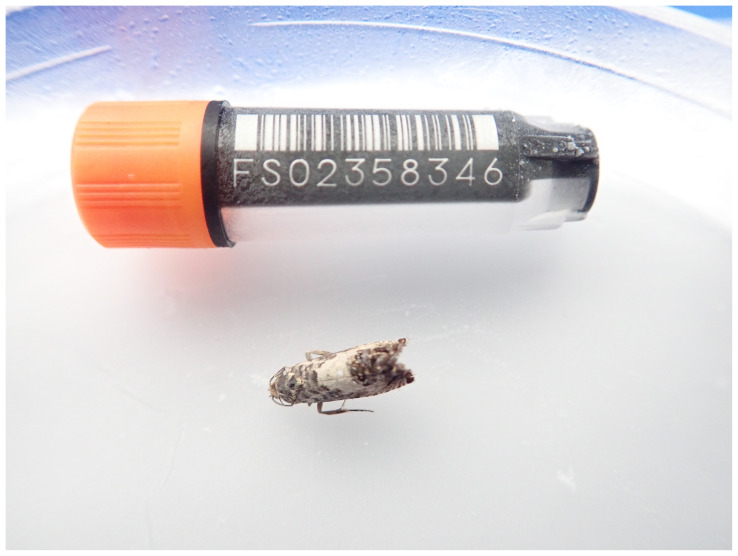
Image of the *Pammene fasciana* specimen (ToLID ilPamFasc1) taken prior to preservation and processing.

The genome was sequenced from a single male *P. fasciana* collected from Wytham Woods, Berkshire, UK ( Figure 1). A total of 36-fold coverage in Pacific Biosciences single-molecule HiFi long reads and 69-fold coverage in 10X Genomics read clouds were generated. Primary assembly contigs were scaffolded with chromosome conformation Hi-C data. Manual assembly curation corrected 11 missing/misjoins, reducing the scaffold number by 19.51%, and increasing the scaffold N50 by 1.19%.

**Figure 2.**
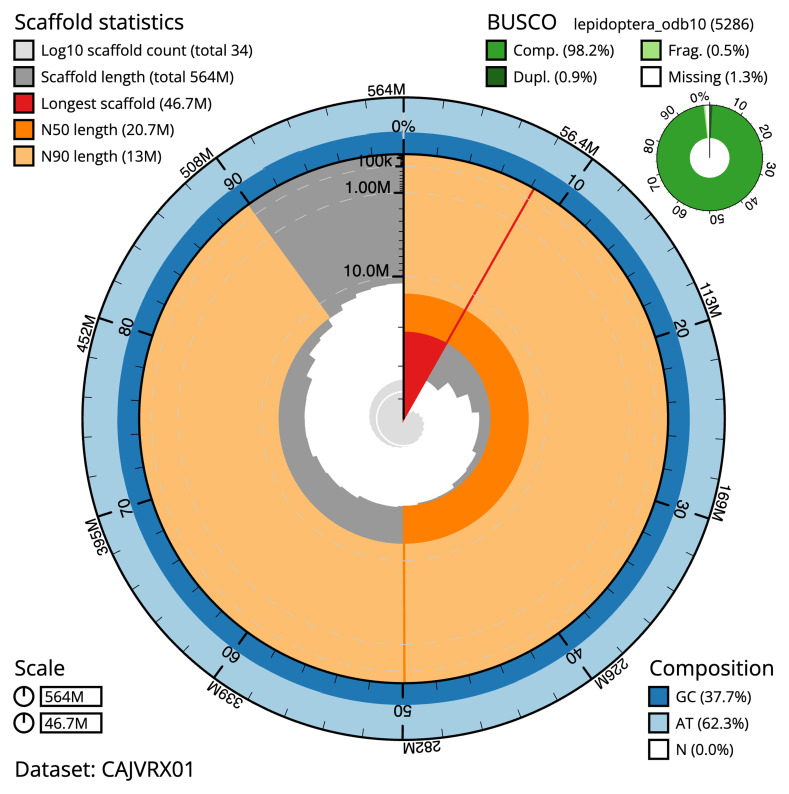
Genome assembly of *Pammene fasciana*, ilPamFasc1.1: metrics. The BlobToolKit Snailplot shows N50 metrics and BUSCO gene completeness. The main plot is divided into 1,000 size-ordered bins around the circumference with each bin representing 0.1% of the 564,437,882 bp assembly. The distribution of chromosome lengths is shown in dark grey with the plot radius scaled to the longest chromosome present in the assembly (46,694,440 bp, shown in red). Orange and pale-orange arcs show the N50 and N90 chromosome lengths (20,742,756 and 12,967,311 bp), respectively. The pale grey spiral shows the cumulative chromosome count on a log scale with white scale lines showing successive orders of magnitude. The blue and pale-blue area around the outside of the plot shows the distribution of GC, AT and N percentages in the same bins as the inner plot. A summary of complete, fragmented, duplicated and missing BUSCO genes in the lepidoptera_odb10 set is shown in the top right. An interactive version of this figure is available at https://blobtoolkit.genomehubs.org/view/ilPamFasc1.1/dataset/CAJVRX01/snail.

The final assembly has a total length of 564 Mb in 33 sequence scaffolds with a scaffold N50 of 20.7 Mb ( Table 1). Most of the assembly sequence (99.94%) was assigned to 28 chromosomal-level scaffolds, representing 27 autosomes (numbered by sequence length) and the Z sex chromosome ( Figure 2– Figure 5; Table 2).

**Table 1.** Genome data for *Pammene fasciana*, ilPamFasc1.1.

*Project accession data*	
Assembly identifier	ilPamFasc1.1
Species	*Pammene fasciana*
Specimen	ilPamFasc1 (genome assembly); ilPamFasc2 (Hi-C)
NCBI taxonomy ID	1101027
BioProject	PRJEB45670
BioSample ID	SAMEA7701530
Isolate information	Male, whole organism (ilPamFasc1); whole organism (ilPamFasc2)
*Raw data accessions*	
PacificBiosciences SEQUEL II	ERR6939220
10X Genomics Illumina	ERR6363300-ERR6363303
Hi-C Illumina	ERR6363304
*Genome assembly*	
Assembly accession	GCA_911728535.1
*Accession of alternate * *haplotype*	GCA_911728525.2
Span (Mb)	564
Number of contigs	71
Contig N50 length (Mb)	16.01
Number of scaffolds	34 (including the mitochondrial sequence)
Scaffold N50 length (Mb)	20.7
Longest scaffold (Mb)	46.7
BUSCO * genome score	C:98.2%[S:97.3%,D:0.9%], F:0.5%,M:1.3%,n:5,286
Genome annotation of assembly GCA_911728535.1 at Ensembl	
Number of protein-coding genes	21,224
Number of gene transcripts	21,492

*BUSCO scores based on the lepidoptera_odb10 BUSCO set using v5.3.2. C= complete [S= single copy, D=duplicated], F=fragmented, M=missing, n=number of orthologues in comparison. A full set of BUSCO scores is available at https://blobtoolkit.genomehubs.org/view/ilPamFasc1.1/dataset/CAJVRX01/busco.

**Figure 3.**
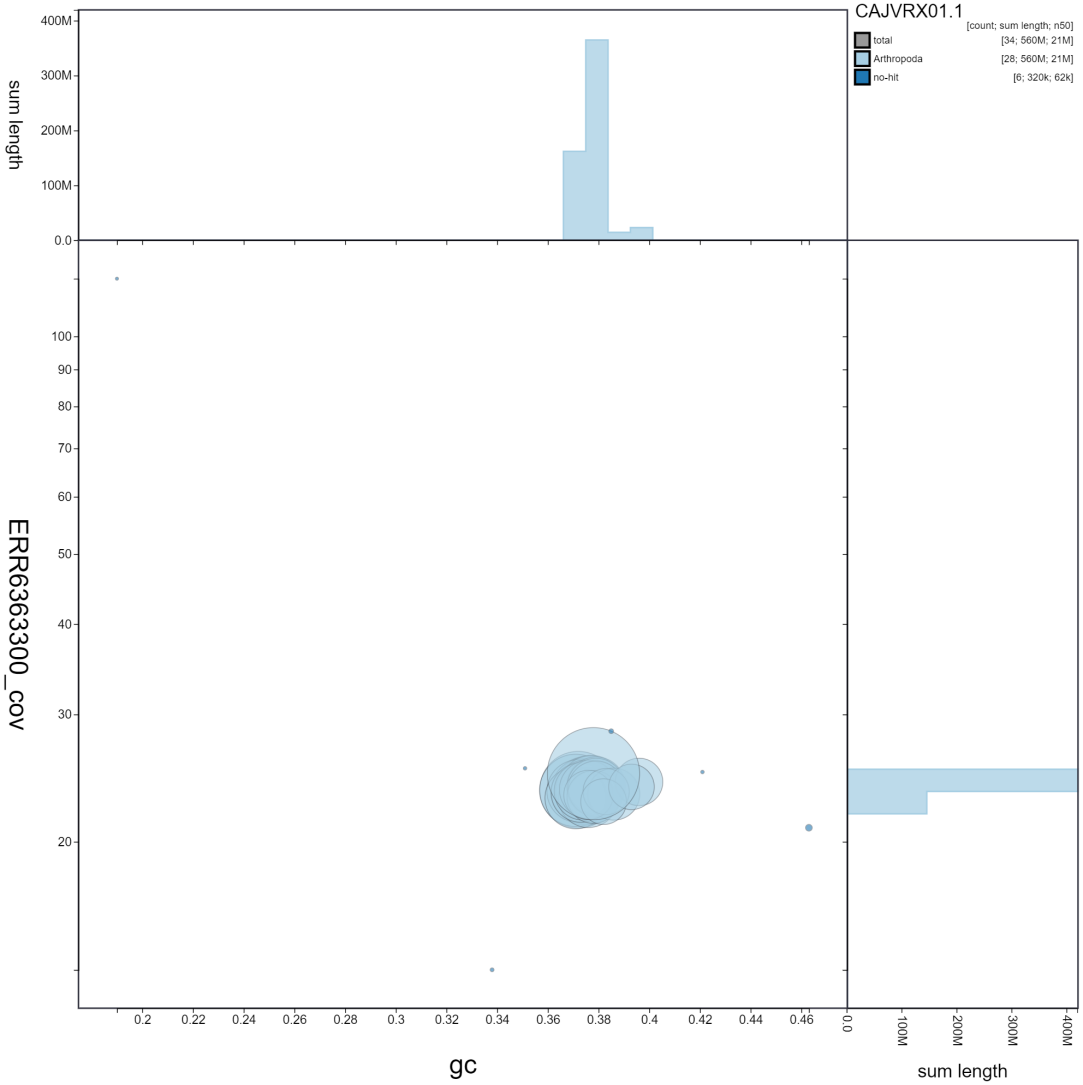
Genome assembly of *Pammene fasciana*, ilPamFasc1.1: BlobToolKit GC-coverage plot showing sequence coverage (vertical axis) and GC content (horizontal axis). The circles represent scaffolds, with the size proportional to scaffold length and the colour representing phylum membership. The histograms along the axes display the total length of sequences distributed across different levels of coverage and GC content. An interactive version of this figure is available at https://blobtoolkit.genomehubs.org/view/ilPamFasc1.1/dataset/CAJVRX01.1/blob.

**Figure 4.**
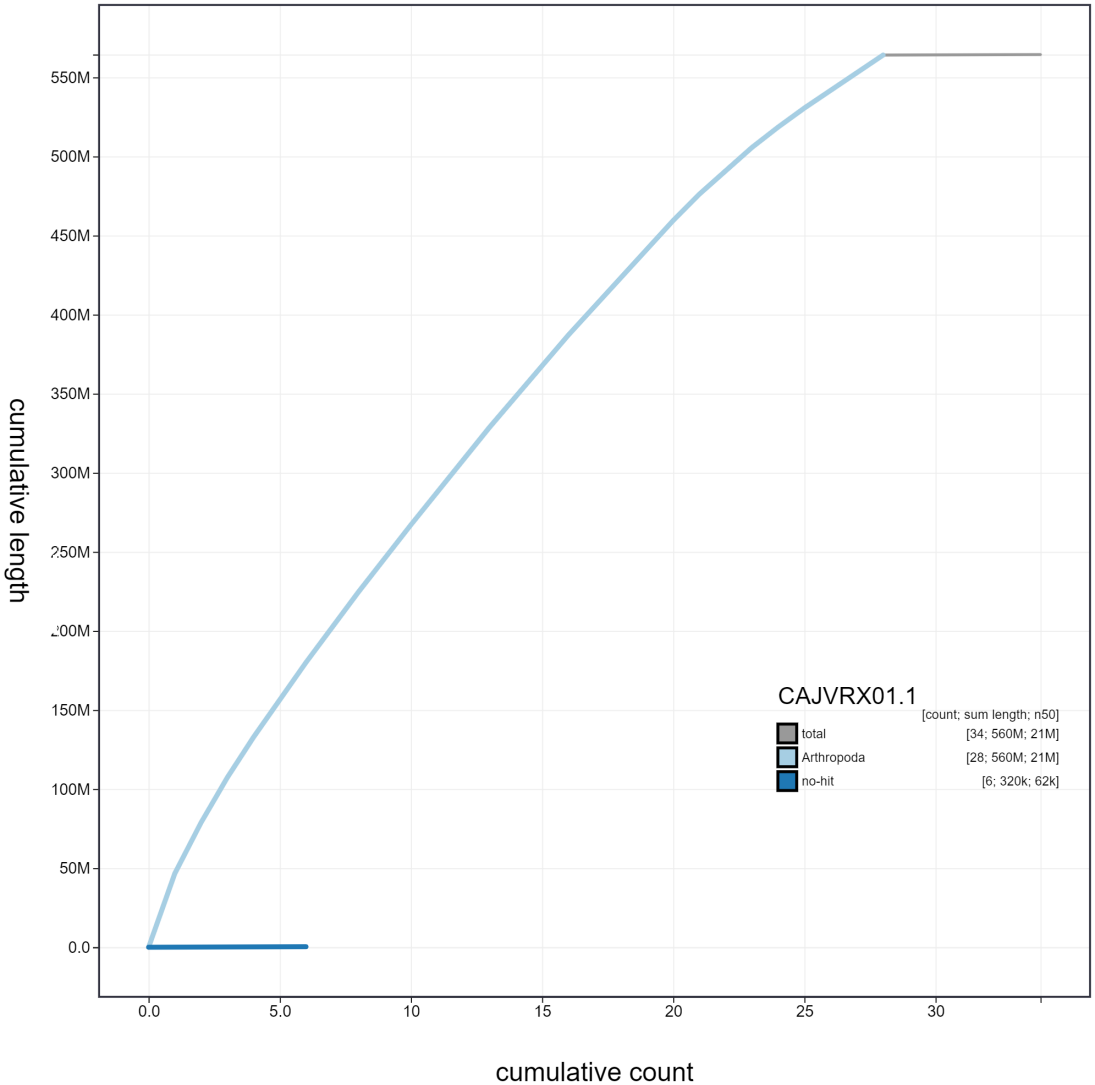
Genome assembly of *Pammene fasciana*, ilPamFasc1.1: cumulative sequence length. BlobToolKit cumulative sequence plot. The grey line shows cumulative length for all scaffolds. Coloured lines show cumulative lengths of scaffolds assigned to each phylum using the buscogenes taxrule. An interactive version of this figure is available at https://blobtoolkit.genomehubs.org/view/ilPamFasc1.1/dataset/CAJVRX01/cumulative.

**Figure 5.**
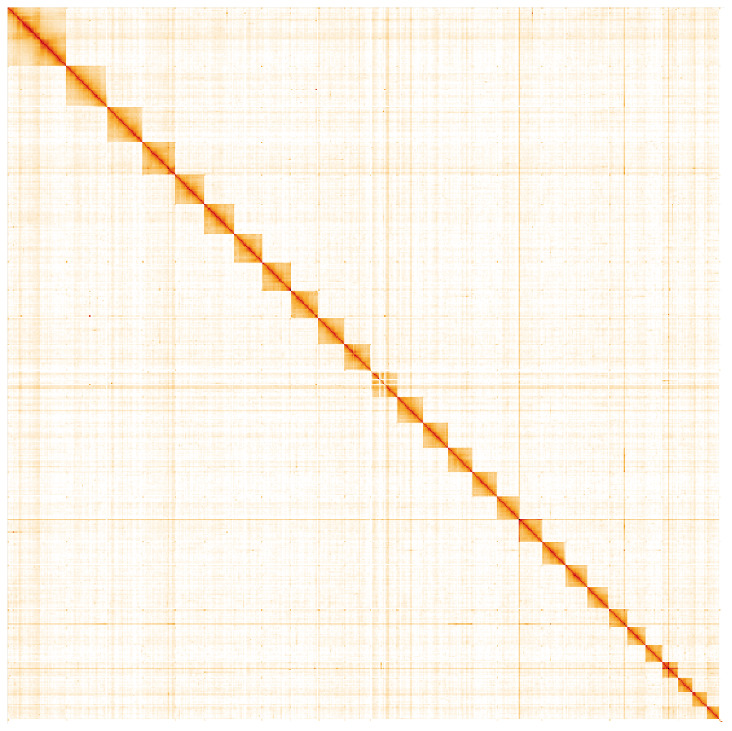
Genome assembly of *Pammene fasciana*, ilPamFasc1.1: Hi-C contact map. Hi-C contact map of the ilPamFasc1.1 assembly, visualised in HiGlass. Chromosomes are arranged in size order from left to right and top to bottom. The interactive Hi-C map can be viewed at https://genome-note-higlass.tol.sanger.ac.uk/l/?d=djOZqsL5QBewlQtNZ2pkBw.

**Table 2.** Chromosomal pseudomolecules in the genome assembly of *Pammene fasciana*, ilPamFasc1.1.

INSDC accession	Chromosome	Size (Mb)	GC%
OU452273.1	1	32.18	37.2
OU452274.1	2	28.49	37.1
OU452275.1	3	25.46	37.5
OU452276.1	4	23.63	37.6
OU452277.1	5	23.62	37.7
OU452278.1	6	22.45	37.6
OU452279.1	7	22.15	37.4
OU452280.1	8	21.27	37.2
OU452281.1	9	21.21	37.7
OU452282.1	10	20.74	37.6
OU452283.1	11	20.5	37.7
OU452284.1	12	20.46	37.1
OU452285.1	13	19.76	37.5
OU452286.1	14	19.38	37.6
OU452287.1	15	19.09	37.3
OU452288.1	16	18.26	37.4
OU452289.1	17	18.14	37.9
OU452290.1	18	18.09	37.6
OU452291.1	19	18.01	37.9
OU452292.1	20	16.62	37.9
OU452293.1	21	14.95	37.8
OU452294.1	22	14.52	38.6
OU452295.1	23	12.97	37.6
OU452296.1	24	12	39.6
OU452297.1	25	11.35	38.3
OU452298.1	26	11.13	38.2
OU452299.1	27	11.02	39.3
OU452272.1	Z	46.69	37.8
OU452300.1	MT	0.02	18.9
-	Unplaced	0.3	42.1

The assembly has a BUSCO v5.3.2 ( Manni *et al.,* 2021) completeness of 98.2% (single 97.3%, duplicated 0.9%) using the lepidoptera_odb10 reference set (n=5,286). While not fully phased, the assembly deposited is of one haplotype. Contigs corresponding to the second haplotype have also been deposited.

## Genome annotation report

The Pammene fasciana genome assembly (GCA_911728535.1) was annotated at the European Bioinformatics Institute (EBI) on Ensembl Rapid Release. The resulting annotation includes 21,492 transcribed mRNAs from 21,224 protein-coding genes ( Table 1; https://rapid.ensembl.org/Pammene_fasciana_GCA_911728535.1/Info/Index). The average transcript length is 7,240.24, and there are 5.34 exons per transcript.

## Methods

### Sample acquisition and nucleic acid extraction

A single male *P. fasciana* specimen (ilPamFasc1) was collected using a light trap from Wytham Woods, Oxfordshire, UK (latitude 51.772, longitude –1.338) by Douglas Boyes (University of Oxford). Specimen ilPamFasc1 was used for DNA sequencing. The specimen used for Hi-C sequencing (ToLID ilPamFasc2) was collected on the same occasion. The specimens were identified by Douglas Boyes, based on morphological examination, and then snap-frozen on dry ice.

DNA was extracted at the Tree of Life Laboratory, Wellcome Sanger Institute. The ilPamFasc1 sample was weighed and dissected on dry ice. Whole organism tissue was disrupted using a Nippi Powermasher fitted with a BioMasher pestle. Fragment size analysis of 0.01–0.5 ng of DNA was then performed using an Agilent FemtoPulse. High molecular weight (HMW) DNA was extracted using the Qiagen MagAttract HMW DNA extraction kit. Low molecular weight DNA was removed from a 200-ng aliquot of extracted DNA using 0.8X AMpure XP purification kit prior to 10X Chromium sequencing; a minimum of 50 ng DNA was submitted for 10X sequencing. HMW DNA was sheared into an average fragment size between 12–20 kb in a Megaruptor 3 system with speed setting 30. Sheared DNA was purified by solid-phase reversible immobilisation using AMPure PB beads with a 1.8X ratio of beads to sample to remove the shorter fragments and concentrate the DNA sample. The concentration of the sheared and purified DNA was assessed using a Nanodrop spectrophotometer and Qubit Fluorometer and Qubit dsDNA High Sensitivity Assay kit. Fragment size distribution was evaluated by running the sample on the FemtoPulse system.

### Sequencing

Pacific Biosciences HiFi circular consensus and 10X Genomics Chromium read cloud sequencing libraries were constructed according to the manufacturers’ instructions. Sequencing was performed by the Scientific Operations core at the Wellcome Sanger Institute on Pacific Biosciences SEQUEL II (HiFi) and Illumina NovaSeq 6000 (10X) instruments. Hi-C data were generated in the Tree of Life Laboratory from whole organism tissue of ilPamFasc2 using the Arima v2 kit and sequenced on a NovaSeq 6000 instrument.

### Genome assembly

Assembly was carried out with Hifiasm ( Cheng *et al.,* 2021); haplotypic duplication was identified and removed with purge_dups ( Guan *et al.,* 2020). One round of polishing was performed by aligning 10X Genomics read data to the assembly with longranger align, calling variants with freebayes ( Garrison & Marth, 2012). The assembly was then scaffolded with Hi-C data ( Rao *et al.,* 2014) using SALSA2 ( Ghurye *et al.,* 2019). The assembly was checked for contamination as described previously ( Howe *et al.,* 2021). Manual curation was performed using HiGlass ( Kerpedjiev *et al.,* 2018) and Pretext. The mitochondrial genome was assembled using MitoHiFi ( Uliano-Silva *et al.,* 2021), which performs annotation using MitoFinder ( Allio *et al.,* 2020). The genome was analysed and BUSCO scores generated within the BlobToolKit environment ( Challis *et al.,* 2020). Table 3 contains a list of all software tool versions used, where appropriate.

**Table 3.** Software tools used.

Software tool	Version	Source
Hifiasm	0.15.1	Cheng *et al.,* 2021
purge_dups	1.2.3	Guan *et al.,* 2020
SALSA2	2.2	Ghurye *et al.,* 2019
longranger align	2.2.2	https://support.10xgenomics.com/ genome-exome/software/pipelines/ latest/advanced/other-pipelines
freebayes	1.3.1-17- gaa2ace8	Garrison & Marth, 2012
MitoHiFi	2.0	Uliano-Silva *et al.,* 2021
HiGlass	1.11.6	Kerpedjiev *et al.,* 2018
PretextView	0.2.x	https://github.com/wtsi-hpag/ PretextView
BlobToolKit	3.2.6	Challis *et al.,* 2020

### Genome annotation

The BRAKER2 pipeline ( Brůna *et al.,* 2021) was used in the default protein mode to generate annotation for the *Pammene fasciana* assembly (GCA_911728535.1) in Ensembl Rapid Release at the EBI.

### Ethics/compliance issues

The materials that have contributed to this genome note have been supplied by a Darwin Tree of Life Partner. The submission of materials by a Darwin Tree of Life Partner is subject to the Darwin Tree of Life Project Sampling Code of Practice. By agreeing with and signing up to the Sampling Code of Practice, the Darwin Tree of Life Partner agrees they will meet the legal and ethical requirements and standards set out within this document in respect of all samples acquired for, and supplied to, the Darwin Tree of Life Project. Each transfer of samples is further undertaken according to a Research Collaboration Agreement or Material Transfer Agreement entered into by the Darwin Tree of Life Partner, Genome Research Limited (operating as the Wellcome Sanger Institute), and in some circumstances other Darwin Tree of Life collaborators.

## Data Availability

European Nucleotide Archive: Pammene fasciana (acorn piercer). Accession number PRJEB45670; https://identifiers.org/ena.embl/PRJEB45670. The genome sequence is released openly for reuse. The *P. fasciana* genome sequencing initiative is part of the Darwin Tree of Life (DToL) project. All raw sequence data and the assembly have been deposited in INSDC databases. Raw data and assembly accession identifiers are reported in Table 1.

## Author information

Members of the University of Oxford and Wytham Woods Genome Acquisition Lab are listed here: https://doi.org/10.5281/zenodo.6418202.

Members of the Darwin Tree of Life Barcoding collective are listed here: https://doi.org/10.5281/zenodo.6418156.

Members of the Wellcome Sanger Institute Tree of Life programme are listed here: https://doi.org/10.5281/zenodo.6866293.

Members of Wellcome Sanger Institute Scientific Operations: DNA Pipelines collective are listed here: https://doi.org/10.5281/zenodo.5746904.

Members of the Tree of Life Core Informatics collective are listed here: https://doi.org/10.5281/zenodo.6125046.

Members of the Darwin Tree of Life Consortium are listed here: https://doi.org/10.5281/zenodo.6418363.
