# Supplementary material for: The genome sequence of the acorn piercer, Pammene fasciana (Linnaeus, 1761)
Source: Wellcome Open Res. 2024 Oct 17;7:258. Originally published 2022 Oct 13. [Version 2] doi: 10.12688/wellcomeopenres.18114.2 (PMC11512137; doi:10.12688/wellcomeopenres.18114.2)
Supplement: Peer review history

# Reviewer report: Reviewer response for version 2

**DOI:** 10.21956/wellcomeopenres.25631.r105759

**Published:** 2024-10-25

**Authors:** Ljiljana Šašić Zorić

**Affiliations:** 1 BioSense Institute, Dr Zorana Đinđića, Novi Sad, Serbia

I have no further comments.

Are sufficient details of methods and materials provided to allow replication by others?

Partly

Is the rationale for creating the dataset(s) clearly described?

Yes

Are the datasets clearly presented in a useable and accessible format?

Yes

Are the protocols appropriate and is the work technically sound?

Yes

Reviewer Expertise:

Population genetics and molecular taxonomy

I confirm that I have read this submission and believe that I have an appropriate level of expertise to confirm that it is of an acceptable scientific standard.

# Reviewer report: Reviewer response for version 1

**DOI:** 10.21956/wellcomeopenres.20085.r55006

**Published:** 2023-03-03

**Authors:** Ljiljana Šašić Zorić

**Affiliations:** 1 BioSense Institute, Dr Zorana Đinđića, Novi Sad, Serbia

“The genome sequence of the acorn piercer, *Pammene fasciana* (Linnaeus, 1761)” is a technical report on the genome assembly of a moth species *Pammene fasciana*. The article is a significant contribution to further research on acorn piercer as an important pest species of oak and sweet chestnut. The sequencing approaches includes Pacific Biosciences HiFi and 10X Genomics sequencing data, with scaffolding using Hi-C data. The applied methodology is suitable and clearly described, as well as technical aspects of genome assembly. However, there are minor inconsistencies that should be resolved prior to final publication.

 Suggestions for revision:

 Genome sequence report

- Please add ID (ilPamFasc1 or ilPamFasc2) for the specimen on Figure 1 since it differs from the ID shown in the figure.
- In the second paragraph, it is stated that 9.94% of the assembly sequence was assigned to 28 chromosomal-level scaffolds, while in the abstract it is 99.94%
- The number of scaffolds in Table 1 is 33 but, based on Figure 2 and results available at https://blobtoolkit.genomehubs.org/view/ilPamFasc1.1/dataset/CAJVRX01/snail, it seems that there are 34 scaffolds. The same applies for Figures 3 and 4. I suppose that one of these corresponds to mitochondrial genome, but please state that clearly.
- Figure 2 is a bit different compared to the interactive version on the link https://blobtoolkit.genomehubs.org/view/ilPamFasc1.1/dataset/CAJVRX01/snail (scale in the interactive version is set to 564 and 46.7 M, while on the figure it is 560M and 47M). The longest scaffold length is 47Mb in Figure 2, while it is 32.2 in Table 1. Please check this.
- Figures 3 and 4 need some technical corrections such as first letter capitalization in the names of axes and gc should also be capitalized.
- Figure 3 caption should be written more clearly: “Genome assembly of *Pammene fasciana*, ilPamFasc1.1: base coverage in ERR6363300 against GC proportion.”
- Please correct Figure 4 caption – “cumulative sequence length” instead of “cumulative sequence”

Methods

- Please add collection data for ilPamFasc2 specimen.
- There is no information on parameters used in data analysis. If the default values were used, please state that clearly or, in case the data analysis follows the procedure previously described in detail, please refer to the source.

Are sufficient details of methods and materials provided to allow replication by others?

Partly

Is the rationale for creating the dataset(s) clearly described?

Yes

Are the datasets clearly presented in a useable and accessible format?

Yes

Are the protocols appropriate and is the work technically sound?

Yes

Reviewer Expertise:

Population genetics and molecular taxonomy

I confirm that I have read this submission and believe that I have an appropriate level of expertise to confirm that it is of an acceptable scientific standard, however I have significant reservations, as outlined above.

# Reviewer report: Reviewer response for version 1

**DOI:** 10.21956/wellcomeopenres.20085.r52901

**Published:** 2022-11-15

**Authors:** Chang-Bae Kim

**Affiliations:** 1 National Genome Information Center, Korea Research Institute of Bioscience and Biotechnology, Daejeon, South Korea

The manuscript by Boyes and Lewin reports the genome sequence of the acorn piercer, *Pammene fasciana*. The genomic data is a welcome addition to the public databases and may provide raw data for future analyses. However, as a reader, I find some basic information missing from the manuscript.

- Please provide the purpose of this study in the last of the Background section.
- Please provide some details on the availability of genomic data from closely related data.
- It is not clear how the species was identified prior to sequencing.

Are sufficient details of methods and materials provided to allow replication by others?

Yes

Is the rationale for creating the dataset(s) clearly described?

Yes

Are the datasets clearly presented in a useable and accessible format?

Yes

Are the protocols appropriate and is the work technically sound?

Yes

Reviewer Expertise:

Bioinformatics for environmental genome and transcriptome by using machine learning

I confirm that I have read this submission and believe that I have an appropriate level of expertise to confirm that it is of an acceptable scientific standard.
